# How does viewing angle affect the perceived accuracy of Batesian mimicry in hoverflies?

**DOI:** 10.1093/beheco/arae054

**Published:** 2024-07-04

**Authors:** Lucy Baker, Chris Taylor, Francis Gilbert, Tom Reader

**Affiliations:** School of Life Sciences, University Park, University of Nottingham, Nottingham NG7 2RD, United Kingdom; School of Life Sciences, University Park, University of Nottingham, Nottingham NG7 2RD, United Kingdom; School of Life Sciences, University Park, University of Nottingham, Nottingham NG7 2RD, United Kingdom; School of Life Sciences, University Park, University of Nottingham, Nottingham NG7 2RD, United Kingdom

**Keywords:** Batesian, hoverfly, Eye of the Beholder; mimicry, Syrphidae

## Abstract

Despite Batesian mimicry often eliciting predator avoidance, many Batesian mimics, such as some species of hoverfly (Syrphidae), are considered to have an “imperfect” resemblance to their model. One possible explanation for the persistence of apparently imperfect mimicry is that human perceptions of mimicry are different from those of natural predators. Natural predators of hoverflies have different visual and cognitive systems from humans, and they may encounter mimics in a different way. For example, whilst humans often encounter hoverflies at rest on vegetation, or in photographs or textbooks, where they are typically viewed from above, natural predators may approach hoverflies from the side or below. To test how viewing angle affects the perception of mimicry, images of mimetic hoverflies and their models (wasps and bees) were shown from different angles in an online survey. Participants were asked to distinguish between the images of models and mimics. The results show that the viewing angle does affect perceived mimicry in some species, although it does not provide a complete explanation for the persistence of imperfect mimicry in nature. The effect is also highly species-specific. This suggests that to understand better how selection has shaped mimetic accuracy in hoverflies and other taxa, further study is required of the viewing angles that predators utilize most commonly in nature.

## Teaser

The angle a hoverfly is seen from effects if it is mistaken for a harmful insect. Hoverflies look like bees or wasps so that predators will avoid them, but this mimicry is often imperfect. We show that the angle from which a hoverfly is seen can affect how accurately it is identified by predators and whether it is mistaken for a wasp or bee. This effect is species-specific, which hints that the angles of approach by predators that are important to the evolution of mimicry vary among hoverflies.

## Introduction

Batesian mimicry is the phenomenon whereby a harmless species resembles a harmful or unprofitable model, either physically or behaviorally, gaining protection from predators (Bates 1862). The more closely a mimic resembles its model, the greater the chance of being mistaken for being unprofitable, and hence, natural selection may favor evolution towards perfect mimicry. However, the extent to which Batesian mimics resemble their models varies considerably, with some mimics being far from perfect (Gilbert 2005). The hoverfly family (Syrphidae) includes many species that can be considered imperfect mimics (Dittrich et al. 1993; Edmunds 2000; Penney et al. 2012) and poor mimics often outnumber good mimics and models (Dittrich et al. 1993). Different species of hoverflies mimic both wasps and bees in their color and patterning.

However, our observations of the existence of imperfect mimicry are probably skewed by human perception. This is problematic for our understanding of the evolution of mimicry, as considered in the Eye of the Beholder hypothesis (Cuthill and Bennett 1993). The hypothesis suggests that human perceptions of mimetic accuracy are not meaningful if the natural predators of the species concerned have different visual and cognitive systems. Humans have often been used as a proxy for real predators in studies of hoverfly mimicry (e.g. Golding et al. 2005; Taylor et al. 2017), but human perceptions of hoverfly mimicry may differ markedly from those of natural predators of hoverflies, such as birds. For example, humans cannot perceive UV patterns, but most birds can (Lind et al. 2013). Whilst there appears to be no UV component in the patterns of many hoverflies and their models (Taylor et al. 2016), it has been noted that the yellow abdominal markings of *Scaeva pyrastri* reflect UV light (Sherratt and Peet-Paré 2017).

Just as sensory and cognitive differences between humans and natural predators can impact perceptions of mimetic accuracy, so might viewing angle. In studies involving humans, mimics (or images of mimics) are typically viewed from above (i.e. a “bird’s eye view”) (Dittrich et al. 1993; Penney et al. 2012; Taylor et al. 2017). However, it is possible that, due to the differing attack methods of natural predators, hoverflies are more commonly viewed from a different angle. For example, a spider with a sit-and-wait predation strategy may view a hoverfly ventrally as it flies overhead. A bird could view a hoverfly from the side as it catches it in flight, or from above as it catches a hoverfly resting on a flower (Dlusski 1984). This could mean that it is not always beneficial for hoverflies to mimic their models accurately from above, as is typically considered in studies of mimicry, but rather ventrally or from the side. It is perhaps surprising then that, to our knowledge, no studies have considered the impact of viewing angle on perceptions of mimicry in hoverflies or any other taxa.

The effect of viewing angle on predation has been tested in some visual signal systems. An example comes from the study of pattern symmetry, which has been shown to affect conspicuousness to predators, with increased symmetry leading to decreased crypsis (Merilaita and Lind 2006). Mascalzoni et al. (2012) showed that chicks can distinguish between symmetrical and asymmetrical patterns even when the pattern has been rotated from the angle on which the chicks were trained. In contrast, the effectiveness of camouflage in clutches of eggs has been shown to depend on the height and angle from which they are viewed by a predator (Hancock et al. 2023). Other examples of the importance of viewing angle come from the study of iridescence, a potentially important component of some animal color patterns (e.g. Pérez De Lanuza and Font (2016), Kjernsmo et al. (2022)). Therefore, viewing angles should also be considered in studies of mimicry.

This study tests whether viewing angle affects perceptions of the accuracy of mimicry in hoverflies. Human participants were asked to identify hoverflies, bees, wasps, and non-mimetic flies from images showing specimens at different viewing angles. Their responses to these questions allowed us to determine if hoverflies were more likely to be mistaken for their models when shown from certain viewing angles. The decision to use humans in this study, rather than a natural predator of hoverflies, was due to the ease of recording human responses compared to those of natural predators and the opportunity to obtain a large sample size. It also removes the need to subject animals to potential stress. The use of humans as a proxy in this study is justified, because humans are a visual predator (McGuire et al. 2006), as are the predators of many mimetic species. Furthermore, the responses of humans in mimicry studies have been shown to correlate with those of avian predators (e.g. Dittrich et al. 1993; Penney et al. 2012; Taylor et al. 2017).

## Methodology

### Insect specimens and photography

**Fig. 1.**
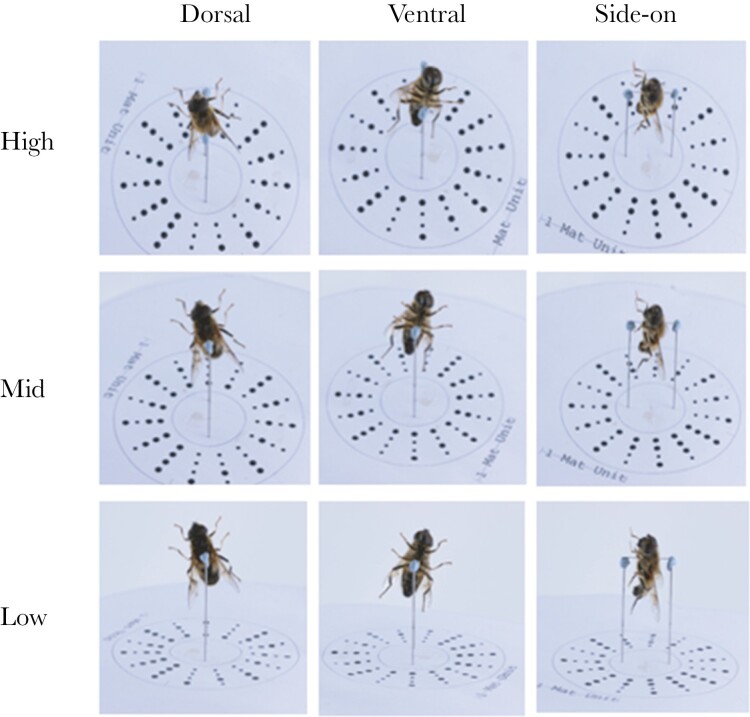
Hoverflies, bees, wasps, and non-mimetic flies were shown from 9 different angle combinations in this study to test whether viewing angle has an effect on the accuracy of identification of those insects. The photos in the figure are examples of those used in the study. Three vertical angles were chosen (high, mid, and low) along with 3 rotational angles (dorsal, ventral, and side-on). This example shows a hoverfly, *E. pertinax*. The pin through the thorax of the hoverfly is 12 mm long.

Insect specimens were collected between June 2020 and August 2021 from various locations in the East Midlands (UK), using a hand net. Species were selected opportunistically from those that were most abundant at our field sites. Specimens were euthanized by freezing at −18 °C for approximately 30 min. They were then pinned through the thorax and positioned into a natural-looking posture before drying for 6–24 h. Specimens were suspended, with the anterior uppermost, on a motorized turntable (Syrp Genie II), positioned against a white background, and lit indirectly using 2 LED panel lights (22 W, 5600 K; Pixapro, Brierley Hill, UK). They were photographed using a DSLR camera (Canon EOS 600D) and macro lens (Tamron SP 90 mm) with F20, 1/6s exposure, ISO400. Each specimen was photographed from 9 different viewing angles—3 different vertical camera positions (vertical angle) at each of 3 equally spaced turntable orientations (rotational angle) (Fig. 1).

Photos of 3 specimens of each of 10 different species were selected for this study. We identified the 3 most abundant aposematic Hymenoptera at our field sites as the most plausible “model” species for hoverfly mimics: *Vespula vulgaris*, *Vespula germanica*, and *Apis mellifera.* The species were divided into a wasp group and a bee group. The wasp group consisted of the 2 common wasp species (*V. vulgaris* and *V. germanica*), 3 wasp mimics (*Epistrophe grossulariae*, *Helophilus pendulus*, and *Sericomyia silentis*) and a control non-mimetic fly (*Mesembrina meridiana*). *E. grossulariae* and *H. pendulus* are both considered accurate mimics of *Vespula* species when observed from a dorsal view (Dittrich et al. 1993; Leavey et al. 2021). The accuracy of *S. silentis* has not been assessed. The bee group consisted of the honeybee, *A. mellifera*, 2 bee mimics (*Eristalis pertinax* and *Eristalis tenax*), and a control non-mimetic fly (*Cheilosia albitarsis*). *Eristalis* species have been shown to be very inaccurate mimics of *Vespula* species when viewed dorsally (Dittrich et al. 1993), but the accuracy of their mimicry of *Apis* has not been quantified. The choice of which control non-mimetic fly was included in each group was arbitrary. A different control non-mimetic fly was included in each group so that all species were encountered the same number of times by each participant (see below).

### Survey

A survey was coded using RStudio (R Core Team 2019) using the shiny (Chang et al. 2022), shinyjs (Attali 2021), shinycssloaders (Sali and Attali 2020), and rdrop2 (Ram and Yochum 2020) packages and deployed on the shinyapps.io server (Posit Software 2022). Upon opening the survey, the user was allocated at random to the bee or wasp group and a single vertical angle and rotational angle combination (from the 9 demonstrated in Fig. 1). The user was given information explaining how to complete the survey (Supplementary Fig. S1), shown a “model photo” (of a specimen that was not included in the question set) of either *A. mellifera* or *V. germanica* (depending on which group had been selected) from the selected viewing angle, and told which model type (“bee” or “wasp”) the photo showed.

The user was then shown the rest of the photos in the group from the selected viewing angle. For the wasp group, participants were shown 18 photos in a random order (3 specimens of 6 species) and asked the question, “Is this a wasp?”. For the bee group, participants were shown 12 photos in a random order (3 specimens of 4 species) and asked the question, “Is this a bee?”. For each image, the participants were asked to select an answer (either “Yes” or “No”), and then were told if they were correct or incorrect. This allowed participants to continue to learn what the images showed throughout the survey. Once all images in the first model group had been shown, the participant was shown the model photo for the other group from the same viewing angle and told which model type it showed. Participants were then asked to classify the images of the species in this group in the same manner as the previous group. The participants were not given a time limit to answer individual questions or complete the survey overall. Once all 30 questions had been asked, the participant was thanked for taking part and shown their overall score.

The link to this survey was sent out to various student and staff email lists at the University of Nottingham, as well as to members of the public via social media. Participants completed the survey using their own device, meaning that display types/resolutions and viewing distances varied. One hundred and forty-six responses to the survey were received. Due to the random allocation to each treatment, the number of participants allocated to each treatment varied from 9 to 20 (*N* for each treatment: high dorsal = 9, high ventral = 17, high side-on = 19, mid dorsal = 16, mid ventral = 20, mid side-on = 18, low dorsal = 18, low ventral = 11, low side-on = 18).

### Statistical analysis

Statistical analysis was carried out using R (version 4.3.1) (R Core Team 2019). A set of nested Generalized Linear Mixed Effects models (GLMMs) were fitted to the entire data set using the glmmML function (Broström 2022). The response variable was whether the image was correctly classified, and binomial errors were assumed. The participant ID and insect specimen were fitted as random effects. The species, vertical angle, rotational angle, and question number were fitted as fixed effects. The order in which the questions were asked was centered around zero, with values ranging from −14.5 to 14.5. Another fixed effect called group order was fitted to account for whether the questions were asked in the first or second group of images. The question number and group order were included in the models to determine if learning occurred across the question set. All 2-way interactions between these fixed effects were included in the maximal model. Preliminary inspection of the data suggested there was not a strong 3-way interaction among species, vertical angle, and rotational angle.

To determine the best model to describe the data, a model selection process was followed as in Symonds and Moussalli (2011) and Smolis et al. (2023). The dredge function from the MuMIn package (Bartoń 2023) was used to fit models with every combination of the fixed effects and their two-way interactions and compare them using the corrected Akaike information criterion (AICc) to find the best model. ΔAICc (the difference in AICc between the best model and model *i*) and the AICc weight (*ωi*) were calculated by the dredge function. The accumulated AICc weight (acc *ωi*) was calculated and used to determine the 95% confidence set of models. The evidence ratio (ER) was calculated using the equation:


$$\begin{array}{l} ER=\frac{exp(-1/2\;\Delta AICc_{best})}{\exp(-1/2\;\Delta AICc_{i})}, \end{array}$$

(from Symonds and Moussalli (2011)) where $\;\Delta \;AICc_{best}$ is the ΔAICc value for the best model, so is equal to zero. The predictor weight was calculated for each predictor by summing the weights of the models in which they were included. In preliminary analysis, we encountered convergence errors for some statistical models, owing to the near-perfect recognition of images of the control (non-mimetic) species, and one of the *A. mellifera* specimens. To avoid this problem, these images were removed from the dataset before conducting the final analysis.

## Results

**Fig. 2.**
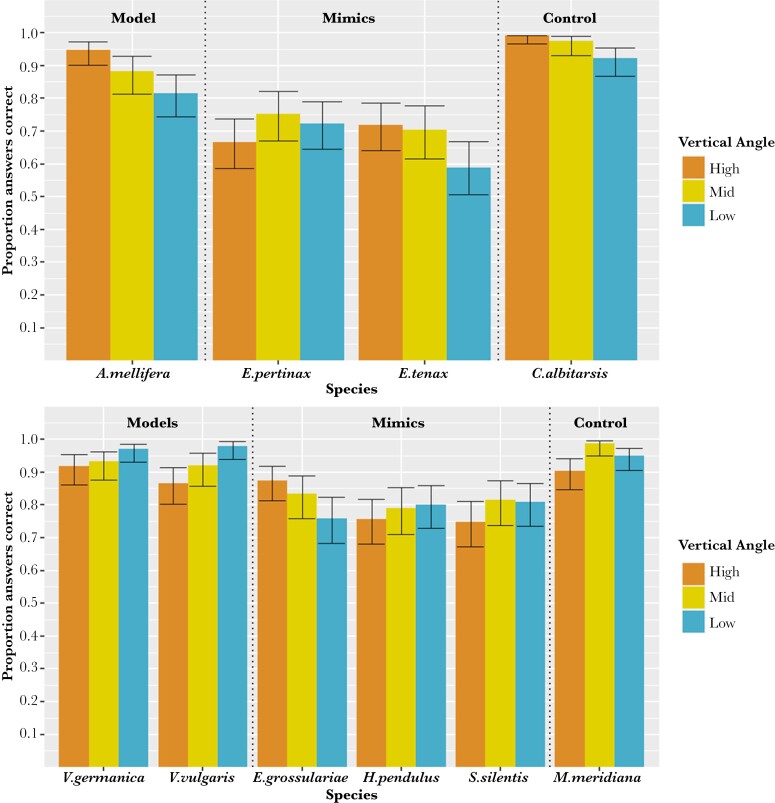
Overall proportion of images of (top) bees, their mimics and non-mimetic flies and (bottom) wasps, their mimics, and non-mimetic flies that were correctly classified when viewed from 3 different vertical angles (high, mid and low, shown by the coloured bars) in an online survey. The total combined responses from all questions asked to every participant are shown. Error bars were calculated using the binomial confidence interval. N for each vertical angle: high = 1350, mid = 1620, low = 1410.

**Fig. 3.**
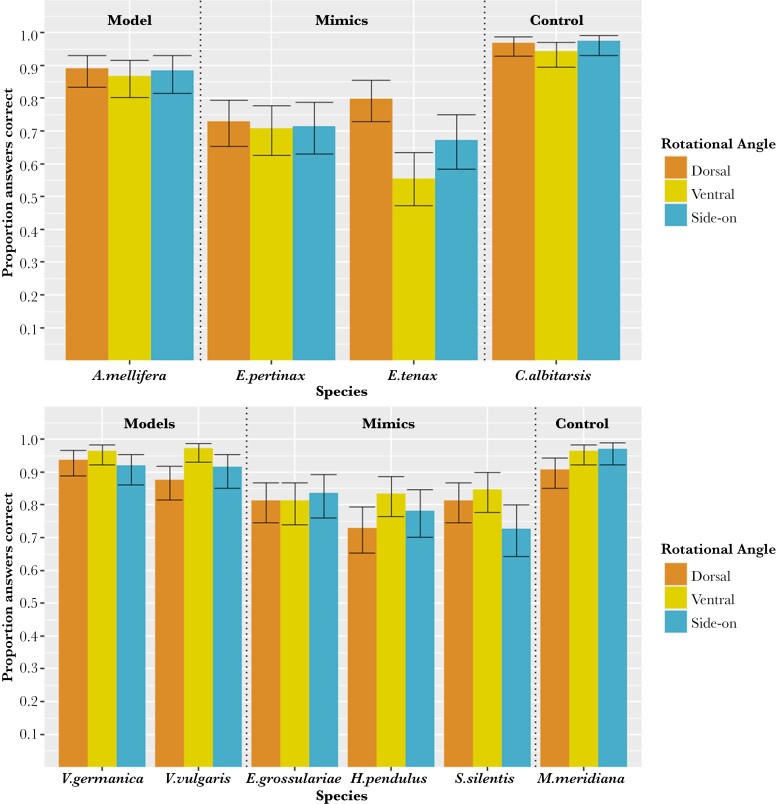
Overall proportion of images of (top) bees, their mimics and non-mimetic flies and (bottom) wasps, their mimics and non-mimetic flies that were correctly classified when observed from 3 different rotational angles (dorsal, ventral and side-on, shown by the coloured bars) in an online survey. The total combined responses from all questions asked to every participant are shown. Error bars were calculated using the binomial confidence interval. *N* for each rotational angle: dorsal = 1,290, side-on = 1,650, ventral = 1,440.

In general, participants were very effective (81% correct or better) at identifying bees, wasps, and non-mimetic fly species (Figs. 2 and 3). Both bee-mimicking and wasp-mimicking hoverflies were identified less accurately, with the honeybee-mimicking *E. tenax* being most likely to be confused with its model. This illustrates that the main effect of species had a strong effect on the accuracy of identification of bees, wasps, hoverflies, and non-mimetic flies. Species were an important predictor in all of the models in the 95% confidence set (Supplementary Table S2).

Ninety-seven models were included in the 95% confidence set (Supplementary Table S2). Due to the low Akaike weights of all the models, no single model can be said to describe the data best, and, therefore, there is strong model selection uncertainty. This is also shown by the ER, which suggests the first model is only 1.89 times more likely to be the best model than the second model. However, the best models shared many terms, indicating that there is a set of predictors that can be considered statistically significant. The predictor weights and coefficients (Supplementary Table S3) show that all the main effects (species, vertical angle, rotational angle, question number, and group order) are important predictors, as well as the interaction between vertical angle and rotational angle, as they all appear in every model in the set and have predictor weights of 0.950. The interactions between species and vertical angle and species and rotational angle also had high predictor weights (0.924 and 0.931, respectively), whilst all other interactions were not well supported (weights < 0.320).

On average, people were most likely to correctly identify the insect shown when it was viewed from the mid-vertical angle (85.93%) and least likely to correctly identify the insect shown when it was viewed from the low vertical angle (83.19 %) (Fig. 2). The interaction between species and vertical angle had a significant effect on the accuracy of identification of bees, wasps, hoverflies, and non-mimetic flies. *Vespula germanica, V. vulgaris,* and their mimic *H. pendulus* were all identified more accurately when viewed from the low vertical angle, whereas *E. pertinax, Mesembrina meridiana,* and *S. silentis* were identified more accurately when viewed from the mid-vertical angle (Fig. 2). *Apis mellifera, E. tenax, C. albitarsis,* and *E. grossulariae* were identified the most accurately when viewed from the high vertical angle.

**Fig. 4.**
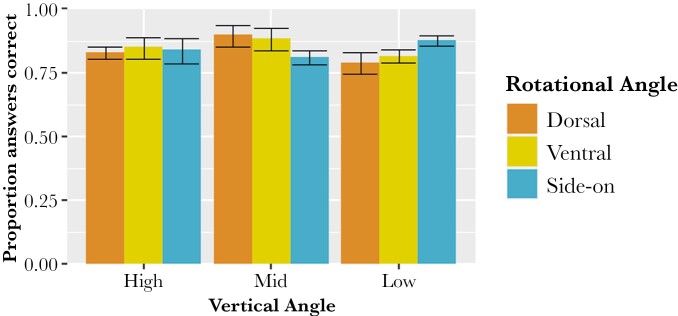
Proportion of correct answers from a survey that asked participants to identify wasps, bees, hoverflies, and non-mimetic files from different viewing angles. The viewing angles consisted of 9 combinations of vertical angle (high, mid, and low, shown on the × axis) and rotational angle (dorsal, ventral and side-on, shown by the coloured bars). The total combined responses from all questions asked to every participant are shown. Error bars were calculated using the binomial confidence interval. *N* for each treatment: High dorsal = 510, high ventral = 360, high side-on = 480, mid dorsal = 480, mid ventral = 480, mid side-on = 660, low dorsal = 300, low ventral = 600, low side-on = 510.

There was a very small main effect of rotational angle on the accuracy of identification of the insects shown, with all 3 rotational angles tested having very similar overall percentage accuracies (dorsal = 84.65%, ventral = 84.72%, side-on = 84.00%) (Figs. 3 and 4). However, rotational angle had a strong interaction with species. *A. mellifera*, and its mimics *E. pertinax* and *E. tenax* were all identified most accurately from a dorsal rotational angle and least accurately from a ventral rotational angle (Fig. 3). In contrast, *V. vulgaris* and *V. germanica* were most accurately identified from a ventral rotational angle, as were their mimics *H. pendulus* and *S. silentis*. However, this was not the case for the wasp mimic *E. grossulariae* which was most accurately identified from a side-on rotational angle.

Rotational angle also had a clear interaction with the vertical angle. Specimens were least accurately identified overall when viewed dorsally and from the low vertical angle (79.00%) and most accurately identified when viewed dorsally from the mid-vertical angle (90.00%) (Fig. 4).

There was a significant effect of question number on the accuracy of the answers given, with a positive trend in accuracy as question number increased. The average accuracy over the first 5 questions was 81.37%, but this increased to 86.30% over the last 5 questions in the question set. Overall, the bee group was more difficult (80.82% accuracy compared to 86.83% accuracy for the wasp group). The order that the groups were asked in (i.e. bee group first or wasp group first) had no effect on the overall accuracy (84.57% for bee group first, 84.30% for wasp group first).

## Discussion

This study has shown that, with humans as a proxy for wild predators, the viewing angle that hoverflies are viewed from affects the accuracy with which they are identified. This could suggest that traditional descriptions of mimetic accuracy and imperfect mimicry, which are typically based on a top-down view of the mimic and model in question, may be flawed. As well as this, we found that hoverflies were consistently identified less accurately than their wasp and bee models. This shows that humans are confused by the appearance of hoverflies and therefore confirms that hoverfly mimicry of their models is effective.

In response to the proposed hypothesis that some apparently imperfect mimics when viewed from above will be more effective mimics when viewed from alternative angles, this study has shown that some hoverfly species are more easily confused with their models when viewed from certain angles. However, this effect is highly species-specific, as shown by the importance of the species × vertical angle and species × rotational angle interactions in the models used to describe the data. The impact of viewing angle on the classification of “prey” is species-specific, and hence may be an important factor in explaining imperfect mimicry in some species, but not others. For example, the ability of study participants to identify *E. tenax* correctly was strongly affected by both vertical angle and rotational angle, with the probability of confusion with the bee model varying from 20% to 40%; by contrast, misclassification of *E. pertinax* was only modestly affected by vertical angle and unaffected by rotational angle.

The misidentification of some hoverflies from certain viewing angles could show a reliance by predators on features of a hoverfly’s appearance for identification that are evolutionarily constrained. Physiological and developmental constraints could stop certain features of a hoverfly’s anatomy, such as their genitals, from evolving to be identical to that of a wasp or bee (Taylor et al. 2017). If said feature is only visible from some angles, this may affect how accurately hoverflies are identified. For example, in this study, the eyes of the insects are easily seen in the images from a high vertical angle, but they become increasingly obscured from the other vertical angles. Alternatively, the effect of the viewing angle may reflect the differing behaviors of different species of hoverfly. For example, where the hoverfly tends to rest or the flowers that it visits may mean that it is more likely to be viewed from a certain angle by predators.

The species specificity of the effect of viewing angle, as well as the relatively weak effect on the data of vertical angle and rotational angle when considered on their own, suggests that it is not possible to generalize about the importance of viewing angle when evaluating mimetic accuracy. Therefore, it cannot be said for certain that viewing angle is an important factor in the Eye of the Beholder hypothesis. To fully assess this, more information on the viewing angles that predators observe hoverflies from needs to be established. Remarkably little is known about the true predators of hoverflies. It is known that spiders and birds, for example, attack hoverflies (Mostler 1935; Dlusski 1984; Gilbert 2005), but it is unclear how important they are in the diet and whether individual predator species prey on specific species of hoverflies. Consequently, we can only guess at which viewing angle predators typically experience when hunting for hoverflies and how this differs between species. Until this is established, the importance of viewing angle in the Eye of the Beholder hypothesis cannot be confirmed.

Determining which angles predators typically view prey from would also help to assess which aspects of an insect’s appearance predators use to identify whether an insect is a palatable and/or profitable prey item. In the photos used in this study, insects shown from the high ventral viewing angle had their eyes, mouthparts, legs, and ventral side of their abdomen and thorax visible. The yellow and black aposematic coloring on the abdomen that is typically used to indicate that a prey item is not palatable was not visible. This is in stark contrast to the mid-dorsal view, where the dorsal side of the thorax and abdomen, including the yellow and black pattern, was visible, but only the top of the eyes could be seen. Therefore, determining what a predator sees when it is attacking a prey item and what features it uses to determine if a prey item is palatable could help to determine if imperfect mimics truly are imperfect from all viewing angles.

It is important to consider the validity of using humans as a proxy in mimicry studies, especially when considering a theory that discusses how their perceptions differ from real-life predators. In this study, overall, the accuracy of identification increased across the question set. This shows that learning occurred throughout the survey, as would be expected from predators attacking hoverflies and their mimics in the wild. It has also been shown that there are strong correlations between human perception and automated or objective measurements of mimetic accuracy (Penney et al. 2012; Taylor et al. 2013) as well as between human and bird ratings of mimetic accuracy in hoverflies (Dittrich et al. 1993; Penney et al. 2012). However, it must also be considered that previous knowledge of the participants may have influenced their answers.

Other limitations of this study include the artificial nature of the photos used, which provide a simplified view compared to what would be experienced in the wild, and the lack of a time limit to complete the survey. In reality, predators would have to pick out the insect from a complicated background in 3D, potentially whilst the insect is in motion and/or under time pressure before it flies off. Whilst viewing an insect in real-time may provide more information to the predator, for example by being able to view it from different angles and assessing behavioral information, information may also be lost, due to things such as motion blur and limited time for information processing (Chittka and Osorio 2007). This is an important factor to consider in future studies that test the Eye of the Beholder hypothesis. Not only must we consider the identity of the signal receiver (predator) but also the circumstances under which the signal is being received.

## Supplementary Material

arae054_suppl_Supplementary_Material

## Data Availability

Analyses reported in this article can be reproduced using the data provided by Baker et al. (2024).
