# Supplementary material for: How does viewing angle affect the perceived accuracy of Batesian mimicry in hoverflies?
Source: Behav Ecol. 2024 Jul 4;35(5):arae054. doi: 10.1093/beheco/arae054 (PMC11259850; doi:10.1093/beheco/arae054)
Supplement: arae054_suppl_Supplementary_Material [file arae054_suppl_supplementary_material.docx]

Figure S1

A screenshot showing the first screen of a survey which was sent out to members of the public asking them to identify wasps, bees, hoverflies and non-mimetic flies from different viewing angles. This screen explains how to complete the survey and shows a control image, in this case of *Apis mellifera*, to provide an example of what the participant would be shown.


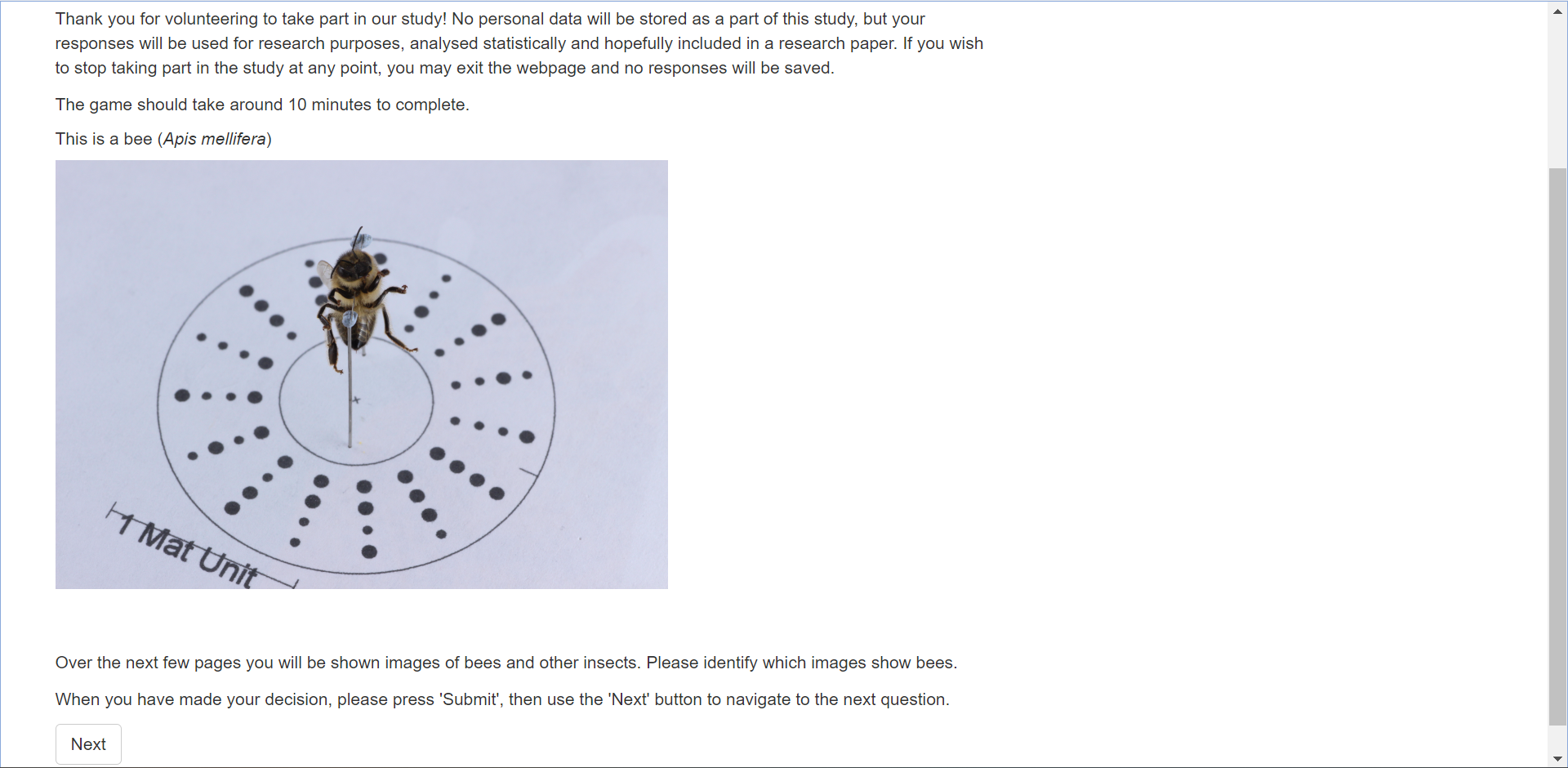


Table S2

95 % confidence set of best ranked candidate Generalized Linear Mixed Effects models examining the effect of species, vertical angle, rotational angle, question number and group order, and two-way interactions between those effects, on the accuracy of identification of bees, wasps, their mimics and non-mimetic flies. The accuracy of identification was assessed using a survey sent out to members of the public where they were asked to identify bees, wasps, hoverflies and non-mimetic flies from photos of specimens shown from different viewing angles. The participants were asked 30 questions in two groups (a “wasp” group, including wasps, wasp-mimicking hoverflies and a control fly and a “bee” group, including a bee, bee mimicking hoverflies and a control fly). k = number of fitted parameters in the model, AICc = Akaike information criterion, ΔAICc = the difference in AICc between the best model and model *i,* ωi = AICc weight, acc ωi = accumulated AICc weight, ER = evidence ratio, S = species of wasp, bee, hoverfly or non-mimetic fly being considered, A = vertical angle the species was viewed from (either high, mid or low), V = rotational angle the species was viewed from (either dorsal, ventral or side-on), Q = question number the species was asked at, centered around zero from -14.5 to 14.5, G = order that the bee and wasp groups were asked in.

|  | Candidate models | k | AICc | ΔAICc | ωi | acc ωi | ER |
| --- | --- | --- | --- | --- | --- | --- | --- |
| 1 | S + A + V + Q + G + S:A + A:V + S:V | 10 | 3022.7 | 0 | 0.147 | 0.147 | 1.00 |
| 2 | S + A + V + Q + G + S:A + A:V + Q:G + S:V | 11 | 3023.9 | 1.27 | 0.078 | 0.225 | 1.89 |
| 3 | S + A + V + Q + G + A:Q + S:A + A:V + S:V | 11 | 3024.5 | 1.82 | 0.059 | 0.284 | 2.48 |
| 4 | S + A + V + Q + G + S:A + A:V + S:G + S:V | 11 | 3025.0 | 2.30 | 0.047 | 0.331 | 3.16 |
| 5 | S + A + V + Q + G + S:A + A:V + V:Q + S:V | 11 | 3025.1 | 2.41 | 0.044 | 0.375 | 3.34 |
| 6 | S + A + V + Q + G + A:G + S:A + A:V + S:V | 11 | 3025.1 | 2.46 | 0.043 | 0.418 | 3.42 |
| 7 | S + A + V + Q + G + S:A + A:V + V:G + S:V | 11 | 3025.4 | 2.75 | 0.037 | 0.455 | 3.96 |
| 8 | S + A + V + Q + G + A:Q + S:A + A:V + Q:G + S:V | 12 | 3025.7 | 3.04 | 0.032 | 0.487 | 4.57 |
| 9 | S + A + V + Q + G + S:A + A:V + Q:G + S:G + S:V | 12 | 3025.8 | 3.19 | 0.03 | 0.517 | 4.93 |
| 10 | S + A + V + Q + G + S:A + A:V + Q:G + V:Q + S:V | 12 | 3026.3 | 3.60 | 0.024 | 0.541 | 6.05 |
| 11 | S + A + V + Q + G + A:G + S:A + A:V + Q:G + S:V | 12 | 3026.4 | 3.72 | 0.023 | 0.564 | 6.42 |
| 12 | S + A + V + Q + G + A:Q + S:A + A:V + V:Q + S:V | 12 | 3026.4 | 3.72 | 0.023 | 0.587 | 6.42 |
| 13 | S + A + V + Q + G + S:A + A:V + Q:G + V:G + S:V | 12 | 3026.7 | 4.03 | 0.02 | 0.607 | 7.50 |
| 14 | S + A + V + Q + G + S:A + A:V + S:G + V:Q + S:V | 12 | 3026.8 | 4.15 | 0.018 | 0.625 | 7.96 |
| 15 | S + A + V + Q + G + A:Q + S:A + A:V + V:G + S:V | 12 | 3026.9 | 4.26 | 0.018 | 0.643 | 8.41 |
| 16 | S + A + V + Q + G + A:Q + S:A + A:V + S:G + S:V | 12 | 3027.2 | 4.52 | 0.015 | 0.658 | 9.58 |
| 17 | S + A + V + Q + G + S:A + A:V + S:G + V:G + S:V | 12 | 3027.2 | 4.52 | 0.015 | 0.673 | 9.58 |
| 18 | S + A + V + Q + G + A:G + S:A + A:V + V:Q + S:V | 12 | 3027.3 | 4.61 | 0.015 | 0.688 | 10.02 |
| 19 | S + A + V + Q + G + A:Q + S:A + A:V + Q:G + V:Q + S:V | 13 | 3027.5 | 4.85 | 0.013 | 0.701 | 11.30 |
| 20 | S + A + V + Q + G + S:A + A:V + Q:G + S:G + V:Q + S:V | 13 | 3027.6 | 4.91 | 0.013 | 0.714 | 11.65 |
| 21 | S + A + V + Q + G + A:G + S:A + A:V + V:G + S:V | 12 | 3027.6 | 4.98 | 0.012 | 0.726 | 12.06 |
| 22 | S + A + V + Q + G + A:G + S:A + A:V + S:G + S:V | 12 | 3028.0 | 5.31 | 0.01 | 0.736 | 14.22 |
| 23 | S + A + V + Q + G + A:Q + S:A + A:V + Q:G + S:G + S:V | 13 | 3028.0 | 5.34 | 0.01 | 0.746 | 14.44 |
| 24 | S + A + V + Q + G + S:A + A:V + Q:G + S:G + V:G + S:V | 13 | 3028.1 | 5.40 | 0.01 | 0.756 | 14.88 |
| 25 | S + A + V + Q + G + A:Q + S:A + A:V + Q:G + V:G + S:V | 13 | 3028.1 | 5.48 | 0.009 | 0.765 | 15.49 |
| 26 | S + A + V + Q + G + A:G + A:Q + S:A + A:V + S:V | 12 | 3028.4 | 5.74 | 0.008 | 0.773 | 17.64 |
| 27 | S + A + V + Q + G + A:G + S:A + A:V + Q:G +V:Q + S:V | 13 | 3028.4 | 5.76 | 0.008 | 0.781 | 17.81 |
| 28 | S + A + V + Q + G + A:Q + S:A + A:V + S:G + V:Q + S:V | 13 | 3028.5 | 5.88 | 0.008 | 0.789 | 18.92 |
| 29 | S + A + V + Q + G + S:A + A:V + S:Q + S:V | 11 | 3028.8 | 6.14 | 0.007 | 0.796 | 21.54 |
| 30 | S + A + V + Q + G + A:G + S:A + A:V + Q:G + S:G + S:V | 13 | 3028.9 | 6.20 | 0.007 | 0.803 | 22.20 |
| 31 | S + A + V + Q + G + A:G + S:A + A:V + Q:G + V:G + S:V | 13 | 3028.9 | 6.23 | 0.007 | 0.81 | 22.53 |
| 32 | S + A + V + Q + G + A:V + S:V | 9 | 3029.0 | 6.36 | 0.006 | 0.816 | 24.05 |
| 33 | S + A + V + Q + G + A:Q + S:A + A:V + S:G + V:G + S:V | 13 | 3029.1 | 6.41 | 0.006 | 0.822 | 24.66 |
| 34 | S + A + V + Q + G + S:A + A:V + V:G + V:Q + S:V | 12 | 3029.1 | 6.43 | 0.006 | 0.828 | 24.90 |
| 35 | S + A + V + Q + G + A:Q + S:A + A:V + Q:G + S:G + V:Q + S:V | 14 | 3029.2 | 6.57 | 0.006 | 0.834 | 26.71 |
| 36 | S + A + V + Q + G + S:A + A:V + Q:G + S:Q + S:V | 12 | 3029.5 | 6.87 | 0.005 | 0.839 | 31.03 |
| 37 | S + A + V + Q + G + A:G + S:A + A:V + S:G + V:Q + S:V | 13 | 3029.6 | 6.98 | 0.004 | 0.843 | 32.79 |
| 38 | S + A + V + Q + G + A:G + A:Q + S:A + A:V + Q:G + S:V | 13 | 3029.7 | 7.02 | 0.004 | 0.847 | 33.45 |
| 39 | S + A + V + Q + G + A:Q + S:A + A:V + Q:G + S:G + V:G + S:V | 14 | 3029.9 | 7.22 | 0.004 | 0.851 | 36.97 |
| 40 | S + A + V + Q + G + A:Q + A:V + S:V | 10 | 3029.9 | 7.24 | 0.004 | 0.855 | 37.34 |
| 41 | S + A + V + Q + G + A:G + S:A + A:V + S:G + V:G + S:V | 13 | 3030.0 | 7.35 | 0.004 | 0.859 | 39.45 |
| 42 | S + A + V + Q + G + A:V + Q:G + S:V | 10 | 3030.1 | 7.48 | 0.003 | 0.862 | 42.10 |
| 43 | S + A + V + Q + G + A:G + A:Q + S:A + A:V + V:Q + S:V | 13 | 3030.3 | 7.64 | 0.003 | 0.865 | 45.60 |
| 44 | S + A + V + Q + G + S:A + A:V + Q:G + V:G + V:Q + S:V | 13 | 3030.3 | 7.66 | 0.003 | 0.868 | 46.06 |
| 45 | S + A + V + Q + G + A:Q + S:A + A:V + V:G + V:Q + S:V | 13 | 3030.4 | 7.73 | 0.003 | 0.871 | 47.70 |
| 46 | S + A + V + Q + G + A:G + S:A + A:V + Q:G + S:G + V:Q + S:V | 14 | 3030.4 | 7.74 | 0.003 | 0.874 | 47.94 |
| 47 | S + A + V + Q + G + S:A + A:V | 9 | 3030.6 | 7.94 | 0.003 | 0.877 | 52.98 |
| 48 | S + A + V + Q + G + A:Q + S:A + A:V | 10 | 3030.6 | 7.94 | 0.003 | 0.88 | 52.98 |
| 49 | S + A + V + Q + G + S:A + A:V + S:Q + V:Q + S:V | 12 | 3030.6 | 7.98 | 0.003 | 0.883 | 54.05 |
| 50 | S + A + V + Q + G + S:A + A:V +S:G + S:Q + S:V | 12 | 3030.7 | 8.08 | 0.003 | 0.886 | 56.83 |
| 51 | S + A + V + Q + G + A:G + S:A + A:V + Q:G + S:G + V:G + S:V | 14 | 3030.9 | 8.22 | 0.002 | 0.888 | 60.95 |
| 52 | S + A + V + Q + G + S:A + A:V + S:G + V:G + V:Q + S:V | 13 | 3030.9 | 8.23 | 0.002 | 0.89 | 61.25 |
| 53 | S + A + V + Q + G + A:G + A:Q + S:A + A:V + V:G + S:V | 13 | 3030.9 | 8.26 | 0.002 | 0.892 | 62.18 |
| 54 | S + A + V + Q + G + A:Q + A:V + Q:G + S:V | 11 | 3031.0 | 8.31 | 0.002 | 0.894 | 63.75 |
| 55 | S + A + V + Q + G + A:G + S:A + A:V | 10 | 3031.0 | 8.33 | 0.002 | 0.896 | 64.39 |
| 56 | S + A + V + Q + G + A:G + A:V + S:V | 10 | 3031.0 | 8.36 | 0.002 | 0.898 | 65.37 |
| 57 | S + A + V + Q + G + A:G + A:Q + S:A + A:V + S:G + S:V | 13 | 3031.0 | 8.37 | 0.002 | 0.9 | 65.69 |
| 58 | S + A + V + Q + G + S:A + A:V + V:G + S:Q + S:V | 12 | 3031.2 | 8.55 | 0.002 | 0.902 | 71.88 |
| 59 | S + A + V + Q + G + S:A + A:V + Q:G + S:Q + V:Q + S:V | 13 | 3031.2 | 8.56 | 0.002 | 0.904 | 72.24 |
| 60 | S + A + V + Q + G + A:Q + S:A + A:V + S:Q + S:V | 12 | 3031.2 | 8.56 | 0.002 | 0.906 | 72.24 |
| 61 | S + A + V + Q + G + A:G + S:A + A:V + V:G + V:Q + S:V | 13 | 3031.3 | 8.66 | 0.002 | 0.908 | 75.94 |
| 62 | S + A + V + Q + G + A:G + A:Q + S:A + A:V + Q:G + V:Q + S:V | 14 | 3031.5 | 8.83 | 0.002 | 0.91 | 82.68 |
| 63 | S + A + V + Q + G + A:Q + S:A + A:V + Q:G + V:G + V:Q + S:V | 14 | 3031.6 | 8.91 | 0.002 | 0.912 | 86.06 |
| 64 | S + A + V + Q + G + A:G + S:A + A:V + S:Q + S:V | 12 | 3031.6 | 8.97 | 0.002 | 0.914 | 88.68 |
| 65 | S + A + V + Q + G + S:A + A:V + Q:G + S:G + V:G + V:Q + S:V | 14 | 3031.7 | 9.03 | 0.002 | 0.916 | 91.38 |
| 66 | S + A + V + Q + G + A:V+ V:Q + S:V | 10 | 3031.7 | 9.07 | 0.002 | 0.918 | 93.22 |
| 67 | S + A + V + Q + G + S:A + A:V + Q:G + S:G + S:Q + S:V | 13 | 3031.8 | 9.13 | 0.002 | 0.92 | 96.06 |
| 68 | S + A + V + Q + G + A:Q + S:A + A:V + Q:G | 11 | 3031.8 | 9.17 | 0.001 | 0.921 | 98.00 |
| 69 | S + A + V + Q + G + A:Q + S:A + A:V + Q:G + S:Q + S:V | 13 | 3031.9 | 9.25 | 0.001 | 0.922 | 102.00 |
| 70 | S + A + V + Q + G + S:A + A:V + Q:G | 10 | 3031.9 | 9.25 | 0.001 | 0.923 | 102.00 |
| 71 | S + A + V + Q + G + A:G + A:Q + S:A + A:V + Q:G + S:G + S:V | 14 | 3031.9 | 9.26 | 0.001 | 0.924 | 102.51 |
| 72 | S + A + V + Q + G + S:A + A:V + Q:G + V:G + S:Q + S:V | 13 | 3031.9 | 9.27 | 0.001 | 0.925 | 103.03 |
| 73 | S + A + V + Q + G + A:Q + S:A + A:V + V:G | 11 | 3031.9 | 9.28 | 0.001 | 0.926 | 103.54 |
| 74 | S + A + V + Q + G + A:V + V:G + S:V | 10 | 3032.0 | 9.36 | 0.001 | 0.927 | 107.77 |
| 75 | S + A + V + Q + G + A:Q + S:A + A:V + V:Q | 11 | 3032.0 | 9.37 | 0.001 | 0.928 | 108.31 |
| 76 | S + A + V + Q + G + A:Q + A:V + V:Q + S:V | 11 | 3032.0 | 9.38 | 0.001 | 0.929 | 108.85 |
| 77 | S + A + V + Q + G + S:A + A:V + V:G | 10 | 3032.1 | 9.42 | 0.001 | 0.93 | 111.05 |
| 78 | S + A + V + Q + G + A:G + A:V + Q:G + S:V | 11 | 3032.1 | 9.46 | 0.001 | 0.931 | 113.30 |
| 79 | S + A + V + Q + G + A:G + A:Q + S:A + A:V + Q:G + V:G + S:V | 14 | 3032.2 | 9.53 | 0.001 | 0.932 | 117.33 |
| 80 | S + A + V + Q + G + S:A + A:V + V:Q | 10 | 3032.3 | 9.61 | 0.001 | 0.933 | 122.12 |
| 81 | S + A + V + Q + G + A:G + S:A + A:V + Q:G | 11 | 3032.3 | 9.62 | 0.001 | 0.934 | 122.73 |
| 82 | S + A + V + Q + G + A:G + S:A + A:V + Q:G + S:Q + S:V | 13 | 3032.4 | 9.70 | 0.001 | 0.935 | 127.74 |
| 83 | S + A + V + Q + G + A:G + A:Q + S:A + A:V + S:G + V:Q + S:V | 14 | 3032.4 | 9.72 | 0.001 | 0.936 | 129.02 |
| 84 | S + A + V + Q + G + S:A + A:V + S:G | 10 | 3032.4 | 9.79 | 0.001 | 0.937 | 133.62 |
| 85 | S + A + V + Q + G + A:G + S:A + A:V + V:G | 11 | 3032.4 | 9.79 | 0.001 | 0.938 | 133.62 |
| 86 | S + A + V + Q + G + A:G + S:A + A:V + Q:G + V:G + V:Q + S:V | 14 | 3032.5 | 9.86 | 0.001 | 0.939 | 138.38 |
| 87 | S + A + V + Q + G + A:Q + A:V + V:G + S:V | 11 | 3032.6 | 9.90 | 0.001 | 0.94 | 141.17 |
| 88 | S + A + V + Q + G + A:Q + S:A + A:V + S:Q + V:Q + S:V | 13 | 3032.6 | 9.94 | 0.001 | 0.941 | 144.03 |
| 89 | S + A + V + Q + G + A:Q + S:A + A:V + S:G + V:G + V:Q + S:V | 14 | 3032.6 | 9.95 | 0.001 | 0.942 | 144.75 |
| 90 | S + A + V + Q + G + A:G + S:A + A:V + V:Q | 11 | 3032.6 | 9.96 | 0.001 | 0.943 | 145.47 |
| 91 | S + A + V + Q + G + S:A + A:V + S:G + S:Q + V:Q + S:V | 13 | 3032.6 | 9.98 | 0.001 | 0.944 | 146.94 |
| 92 | S + A + V + Q + G + A:V + S:G + S:V | 10 | 3032.7 | 10.02 | 0.001 | 0.945 | 149.90 |
| 93 | S + A + V + Q + G + A:V + Q:G + V:Q + S:V | 11 | 3032.7 | 10.08 | 0.001 | 0.946 | 154.47 |
| 94 | S + A + V + Q + G + A:Q + S:A + A:V + S:G | 11 | 3032.9 | 10.28 | 0.001 | 0.947 | 170.72 |
| 95 | S + A + V + Q + G + S:A + A:V + S:G + V:G + S:Q + S:V | 13 | 3033.0 | 10.31 | 0.001 | 0.948 | 173.30 |
| 96 | S + A + V + Q + G + A:Q + A:V + Q:G + V:Q + S:V | 12 | 3033.0 | 10.35 | 0.001 | 0.949 | 176.80 |
| 97 | S + A + V + Q + G + A:G + A:Q + S:A + A:V + S:G + V:G + S:V | 14 | 3033.0 | 10.39 | 0.001 | 0.95 | 180.37 |

Table S3

Predictor weights, coefficients and SEM for each independent variable included in the Generalized Linear Mixed Effects models examining the effect of species, vertical angle, rotational angle, question number and group order, and two-way interactions between those effects, on the accuracy of identification of bees, wasps, their mimics and non-mimetic flies. The accuracy of identification was assessed using a survey sent out to members of the public where they were asked to identify bees, wasps, hoverflies and non-mimetic flies from photos of specimens shown from different viewing angles. The participants were asked 30 questions in two groups (a “wasp” group, including wasps, wasp-mimicking hoverflies and a control fly and a “bee” group, including a bee, bee mimicking hoverflies and a control fly). Predictor weights were calculated by summing the AICc weight (ωi) (Table S2) of each model that the predictor appears in. Coefficients and SEM were calculated using the model.avg function of the MuMIn package (Barton, 2023). S = species of wasp, bee, hoverfly or non-mimetic fly being considered, A = vertical angle the species was viewed from (either high, mid or low), V = rotational angle the species was viewed from (either dorsal, ventral or side-on), Q = question number the species was asked at, centered around zero from -14.5 to 14.5, G = order that the bee and wasp groups were asked in. Intercept represents the coefficients for the default factor levels in the model. These are *A. mellifera* (S), high (A), dorsal (V) and first (G).

| Predictor | Predictor weight | Parameter | Coefficient | SEM |
| --- | --- | --- | --- | --- |
| Intercept |  |  | 2.759 | 0.538 |
| S | 0.950 | *E. grossulariae* | -0.570 | 0.593 |
|  |  | *E. pertinax* | -1.615 | 0.595 |
|  |  | *E. tenax* | -0.894 | 0.599 |
|  |  | *H. pendulus* | -1.509 | 0.581 |
|  |  | *S. silentis* | -1.126 | 0.594 |
|  |  | *V. germanica* | -0.016 | 0.668 |
|  |  | *V. vulgaris* | -0.925 | 0.637 |
| A | 0.950 | Low | -1.482 | 0.564 |
|  |  | Mid | -0.179 | 0.542 |
| V | 0.950 | Ventral | 0.125 | 0.488 |
|  |  | Side | 0.137 | 0.467 |
| Q | 0.950 |  | 0.057 | 0.019 |
| G | 0.950 | Second | -0.787 | 0.390 |
| S:A | 0.924 | Low : *E. grossulariae* | 0.370 | 0.575 |
|  |  | Mid : *E. grossulariae* | 0.377 | 0.590 |
|  |  | Low : *E. pertinax* | 1.460 | 0.613 |
|  |  | Mid : *E. pertinax* | 1.099 | 0.588 |
|  |  | Low : *E. tenax* | 0.741 | 0.556 |
|  |  | Mid : *E. tenax* | 0.698 | 0.564 |
|  |  | Low : *H. pendulus* | 1.324 | 0.616 |
|  |  | Mid : *H. pendulus* | 0.821 | 0.580 |
|  |  | Low : *S. silentis* | 1.481 | 0.630 |
|  |  | Mid : *S. silentis* | 1.197 | 0.612 |
|  |  | Low : *V.germanica* | 2.160 | 0.876 |
|  |  | Mid : *V. germanica* | 0.939 | 0.689 |
|  |  | Low : *V. vulgaris* | 2.905 | 0.987 |
|  |  | Mid : *V. vulgaris* | 1.154 | 0.666 |
| S:V | 0.931 | *E.grossulariae* : ventral | 0.059 | 0.539 |
|  |  | *E.pertinax :* ventral | -0.215 | 0.518 |
|  |  | *E.tenax :* ventral | -1.165 | 0.567 |
|  |  | *H.pendulus :* ventral | 0.519 | 0.542 |
|  |  | *S.silentis* : ventral | 0.133 | 0.549 |
|  |  | *V.germanica :* ventral | 0.283 | 0.741 |
|  |  | *V.vulgaris* : ventral | 1.288 | 0.774 |
|  |  | *E.grossulariae* : side | 0.284 | 0.530 |
|  |  | *E.pertinax* : side | -0.043 | 0.501 |
|  |  | *E tenax* : side | -0.618 | 0.521 |
|  |  | *H.pendulus* : side | 0.346 | 0.513 |
|  |  | *S.silentis* : side | -0.465 | 0.524 |
|  |  | *V.germanica* : side | -0.240 | 0.657 |
|  |  | *V.vulgaris* : side | 0.517 | 0.598 |
| S:Q | 0.034 | *E.grossulariae :* Q | 0.001 | 0.013 |
|  |  | *E.pertinax* : Q | 0.000 | 0.013 |
|  |  | *E.tenax* : Q | 0.001 | 0.013 |
|  |  | *H.pendulus* : Q | 0.003 | 0.018 |
|  |  | *S.silentis :* Q | 0.001 | 0.013 |
|  |  | *V.germanica* : Q | 0.002 | 0.013 |
|  |  | *V.vulgaris* : Q | 0.002 | 0.017 |
| S:G | 0.232 | *E.grossulariae* : second | -0.158 | 0.411 |
|  |  | *E.pertinax :* second | -0.182 | 0.429 |
|  |  | *E.tenax* : second | -0.164 | 0.414 |
|  |  | *H.pendulus* : second | -0.081 | 0.372 |
|  |  | *S.silentis* : second | -0.143 | 0.390 |
|  |  | *V.germanica* : second | 0.192 | 0.476 |
|  |  | *V.vulgaris* : second | -0.033 | 0.368 |
| A:V | 0.950 | Low : ventral | 0.258 | 0.303 |
|  |  | Mid : ventral | -0.252 | 0.314 |
|  |  | Low : side | 0.659 | 0.294 |
|  |  | Mid : side | -0.908 | 0.274 |
| A:Q | 0.254 | Low : Q | -0.006 | 0.013 |
|  |  | Mid : Q | -0.002 | 0.009 |
| A:G | 0.174 | Low : second | -0.052 | 0.173 |
|  |  | Mid : second | -0.030 | 0.138 |
| V:Q | 0.222 | Ventral : Q | 0.001 | 0.008 |
|  |  | Side : Q | -0.003 | 0.009 |
| V:G | 0.178 | Ventral : second | 0.007 | 0.134 |
|  |  | Side : second | -0.041 | 0.148 |
| Q:G | 0.318 | Q : second | 0.007 | 0.016 |

References

Barton, K. (2023) MuMIn: Multi-Model Inference.
